# Singlet Oxygen Metabolism: From Genesis to Signaling

**DOI:** 10.3389/fpls.2019.01640

**Published:** 2020-01-08

**Authors:** Vivek Dogra, Chanhong Kim

**Affiliations:** Shanghai Center for Plant Stress Biology and Center of Excellence in Molecular Plant Sciences, Chinese Academy of Sciences, Shanghai, China

**Keywords:** singlet oxygen (^1^O_2_), grana core (GC), grana margin (GM), photoinhibition, retrograde signaling, β-carotene, EXECUTER1 (EX1)

## Abstract

Singlet oxygen (^1^O_2_) is an excited state of molecular oxygen with an electron spin shift in the molecular orbitals, which is extremely unstable and highly reactive. In plants, ^1^O_2_ is primarily generated as a byproduct of photosynthesis in the photosystem II reaction center (PSII RC) and the light-harvesting antenna complex (LHC) in the grana core (GC). This occurs upon the absorption of light energy when the excited chlorophyll molecules in the PSII transfer the excess energy to molecular oxygen, thereby generating ^1^O_2_. As a potent oxidant, ^1^O_2_ promotes oxidative damage. However, at sub-lethal levels, it initiates chloroplast-to-nucleus retrograde signaling to contribute to plant stress responses, including acclimation and cell death. The thylakoid membranes comprise two spatially separated ^1^O_2_ sensors: β-carotene localized in the PSII RC in the GC and the nuclear-encoded chloroplast protein EXECUTER1 (EX1) residing in the non-appressed grana margin (GM). Finding EX1 in the GM suggests the existence of an additional source of ^1^O_2_ in the GM and the presence of two distinct ^1^O_2_-signaling pathways. In this review, we mainly discuss the genesis and impact of ^1^O_2_ in plant physiology.

## Singlet Oxygen Genesis in PSII-LHC

**Figure 1:**
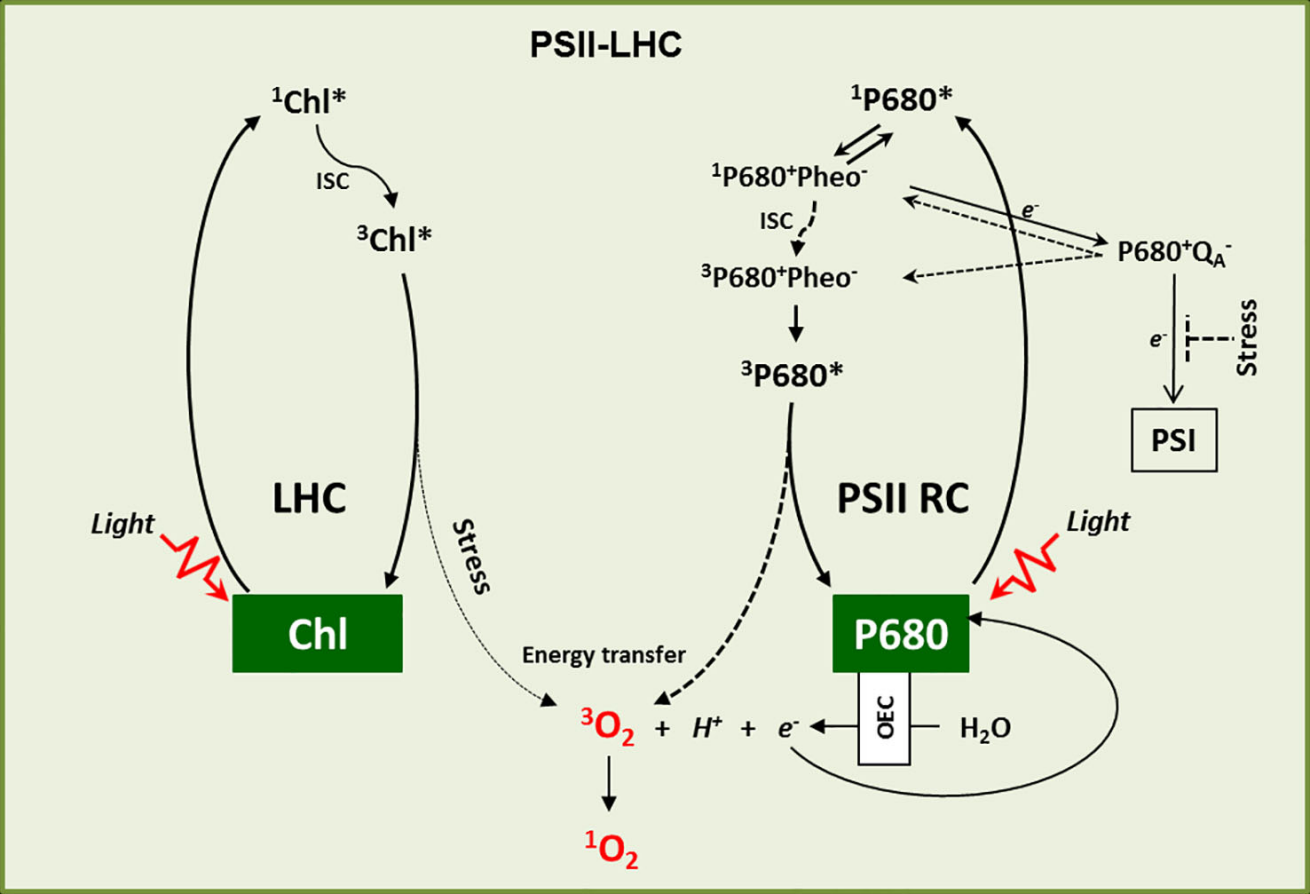
^1^O_2_ genesis. ^1^O_2_ is primarily generated in the LHC and PSII RC. In the LHC, upon absorption of light energy, Chl is excited from the ground state to its excited singlet state (^1^Chl*), which by intersystem crossing (ISC) turns into a comparatively long-lived excited triplet state (^3^Chl*). ^3^Chl* is then quenched by carotenoids to come down to its ground state. However, under high light stress conditions it may react with ground state triplet oxygen (^3^O_2_), which leads to its excited singlet state (^1^O_2_). Similarly, P680 in the PSII RC attains excited singlet state (^1^P680*) upon light absorption. The charge separation reaction between ^1^P680* and Pheophytin (Pheo) results in the formation of the first radical pair ^1^P680^+^Pheo^-^, followed by electron transfer to quinone A (Q_A_) and the formation of the second radical pair ^1^P680^+^Q_A_^−^. After donating its electron to Q_A_, the oxidized P680^+^ is re-reduced through the water-splitting reactions of the oxygen-evolving complex (OEC). However, when the photosynthetic electron transport chain (PETC) is over-reduced, the radical pair P680^+^Q_A_^−^ favors the charge recombination reactions to give rise either to ^3^P680^+^Pheo^-^ through spin reversal or to ^1^P680^+^Pheo^-^. The recombined radical pair ^1^P680^+^Pheo^-^ then decays into ^3^P680^+^Pheo^-^ *via* intersystem crossing. P680 dissociates from ^3^P680^+^Pheo^-^ and subsequently forms ^3^P680*, which reacts with ^3^O_2_ to form ground state P680 and ^1^O_2_. Under stress conditions such as HL, cold, and drought, when the electron acceptor Q_A_ is highly reduced, charge recombination reactions stimulate the accumulation of ^3^P680* and the production of ^1^O_2_.

Singlet oxygen (^1^O_2_) is a peculiar reactive oxygen species (ROS), generated *via* energy transfer from excited chlorophyll to molecular oxygen during photosynthesis mainly at the photosystem II light-harvesting antenna complex (PSII-LHC) located in the appressed region, namely the grana core (GC) of the thylakoid membrane (Foote, 1968; Gollnick, 1968; Krieger‐Liszkay, 2005; Triantaphylidès and Havaux, 2009). Upon absorption of light energy, chlorophyll in LHC attains a high energy but short-lived (few ns, ~10^-8^ s) singlet excited state (^1^Chl*). A part of this absorbed light energy in ^1^Chl* is transferred to the reaction center chlorophyll P680 *via* resonance energy transfer to drive the photosynthetic electron transport chain (PETC). This process is referred to as photochemical quenching as it converts the harvested light energy into chemical energy (Demmig-Adams and Adams, 2000; Muller et al., 2001). However, chlorophyll molecules absorb the light energy that exceeds the capacity of photochemical quenching. To avoid unwanted consequences, this excess of non-utilized light energy from ^1^Chl* is dissipated either as heat (called non-photochemical quenching, NPQ) or as fluorescence (Demmig-Adams and Adams, 2000; Muller et al., 2001). In addition, the energy from ^1^Chl* also gets decayed *via* intersystem crossing (ISC, changing of spin in the molecular orbitals) which results in the formation of a lower energy triplet excited state of chlorophyll (^3^Chl*) with a comparatively longer half-life (~10^-3^ s) (Muller et al., 2001). The carotenoids present in the LHC, such as lutein and zeaxanthin, quench this ^3^Chl* to prevent any unusual transfer of energy to other nearby molecules. However, if this ^3^Chl* is not efficiently quenched, it reacts with molecular oxygen (^3^O_2_) released from the water-splitting reaction in the oxygen-evolving complex (OEC) and leads to the generation of ^1^O_2_ (van Mieghem et al., 1995; Rinalducci et al., 2004; Santabarbara et al., 2007; Li et al., 2009) (**Figure 1**). This gain of energy results in an electron spin shift in the molecular orbitals, which makes ^1^O_2_ very unstable and highly reactive.

In the PSII reaction center (RC), the P680 excites to a singlet state (^1^P680*) once it absorbs light energy. ^1^P680* then forms a radical pair with pheophytin (Pheo), ^1^P680^+^Pheo^-^, the first electron carrier intermediate, through a charge separation reaction (Vass et al., 1992; Adir et al., 2003; Hideg, 2004). This radical pair then transfers an electron to the primary electron acceptor quinone (Q_A_), leading to the formation of the long-lived second radical pair P680^+^Q_A_^−^ (Krieger-Liszkay, 2005). After donating its electron to Q_A_, the oxidized P680^+^ is re-reduced by extracting electrons from the water-splitting reaction in the OEC and returns to its ground state. However, if Q_A_ is reduced due to the blockage of downstream electron transport (also called the closed state of PSII RC), it cannot accept any further electrons (Durrant et al., 1990). Such a condition allows the recombination of the primary radical pair (^3^P680^+^Pheo^-^) with P680 to a triplet state (Durrant et al., 1990; Krieger-Liszkay, 2005). The charge recombination reaction may also result in the formation of ^1^P680^+^Pheo^-^ which consequently decays into ^3^P680^+^Phe^-^ *via* ISC (Durrant et al., 1990; Krieger-Liszkay, 2005). The ^3^P680^+^Pheo^-^ subsequently dissociates into Pheo and ^3^P680* (**Figure 1**). Although PSII RC carries two molecules of β-carotene, their proximity to P680 is more than the Van der Waal’s distance of 3.6 Å, which is essential to quench ^3^P680*. This allows ^3^P680* to react with ^3^O_2_ to generate ^1^O_2_ (Durrant et al., 1990; van Mieghem et al., 1995; Hideg et al., 1998; Krieger-Liszkay, 2005; Santabarbara et al., 2007; Krieger-Liszkay et al., 2008) (**Figure 1**).

## ^1^O_2_ Increases Upon Photo Inhibition

Under long-lasting or adverse light-stress conditions, the rate of ^1^O_2_ generation exceeds the scavenging capacity of chloroplasts and results in the increased photodamage of PSII and consequently decreased photosynthetic efficiency, a phenomenon called photoinhibition, (Hideg et al., 1998). Excess light enhances the rate of electron transfer in PSII, which can surpass the capacity of downstream electron acceptors in the photosynthetic electron transport chain, resulting in the over-reduction of Q_A_ (Vass et al., 1992). The reduced Q_A_ enhances the generation of ^1^O_2_ *via* charge recombination reactions (Vass et al., 1992; Adir et al., 2003; Hideg, 2004) (**Figure 1**). Interestingly, not only the light-absorbing chlorophyll molecules in PSII but also the uncoupled or weakly coupled ones of the antenna complex generate ^1^O_2_, thereby eventually inducing photoinhibition (Santabarbara et al., 2002). Besides, inactivation of the oxygen-evolving complex also leads to photoinhibition, referred to as donor-side photoinhibition (Keren et al., 1997). Plants grown under low light intensities can also exhibit photo-inactivation of PSII with an increased level of ^1^O_2_, upon exposure to a flashlight (Szilard et al., 2005). In the presence of light, other stress factors (such as drought, cold, heat, salinity, and heavy metals) entail photoinhibition as a result of over-reduction of the photosynthetic electron transport chain (PETC) (Foyer and Noctor, 2005; Nishiyama et al., 2006; Takahashi and Murata, 2008; Li et al., 2009; Rochaix, 2011; Foyer et al., 2012; Nishiyama and Murata, 2014). The combination of HL with low temperature leads to photoinhibition as the low temperature slows down the PSII repair process (Baker, 1996). Treatments of plants with various herbicides also result in photoinhibition (Metz et al., 1986; Fufezan et al., 2002). The herbicide diuron (DCMU), e.g., blocks the electron transfer between Q_A_ and Q_B_ of PSII causing photoinhibition *via* ^1^O_2_ (Metz et al., 1986).

## PSII Damage

Being a strong oxidant, ^1^O_2_ induces irreversible photo-oxidative damage to the nearby biomolecules such as proteins, carotenoids, and lipids (Ravanat et al., 2000; Apel and Hirt, 2004; Davies, 2004; Watabe et al., 2007). Prime targets for ^1^O_2_-driven chemical reactions are double bonds, e.g., in aromatic amino acids of proteins, polyunsaturated fatty acids (PUFA) in lipids and guanine bases in DNA, and thiol groups (Buettner, 1993). Besides, scavengers containing alternate double bonds or thiol groups, such as β-carotene, ascorbate, tocopherol, and glutathione, can also undergo oxidations upon a ^1^O_2_ burst. Among these biomolecules, oxidation of lipids by ^1^O_2_ primarily leads to a non-enzymatic peroxidation of PUFA, a hallmark of photo-oxidative damage (Mueller et al., 2006; Przybyla et al., 2008). Likewise, those amino acids containing side chains with alternate double bonds such as cysteine, histidine, methionine, tryptophan, tyrosine, and phenylalanine, all undergo oxidation by ROS (Michaeli and Feitelson, 1994). Tryptophan and its derivatives quench most of the ^1^O_2_, followed by histidine, cysteine, methionine, tyrosine, and phenylalanine (Michaeli and Feitelson, 1994; Davies, 2004).

To alleviate photodamage, ^1^O_2_ needs to be quenched by some molecular components in PSII either by energy transfer or through oxidative modifications (Foote and Denny, 1968; Gorman and Rodgers, 1992; Krieger-Liszkay and Trebst, 2006). Carotenoids, such as lutein and zeaxanthin residing in the close vicinity to Chl in LHC (less than the van der Waals distance of 3.6 Å), directly quench the excited ^3^Chl* and ^1^O_2_ (Gorman and Rodgers, 1992; Telfer et al., 1994; Telfer, 2002; Krieger-Liszkay and Trebst, 2006). This physical quenching of ^3^Chl*/^1^O_2_ excites the carotenoids to a higher energy triplet state (^3^Car*), which then undergoes a decay *via* ISC, triplet-triplet annihilation, or ground-state quenching (Edge and Truscott, 1999; Burke et al., 2000). In contrast to the carotenoids in LHC, the β-carotene molecules present in the PSII RC are situated far from ^3^P680* which prevents their direct quenching (Telfer et al., 1994). However, the distance between β-carotene and the ^1^O_2_ generated in PSII RC is close enough for chemical quenching, leading to the oxidative modification of β-carotene (Foote and Denny, 1968; Krieger-Liszkay, 2005; Ramel et al., 2012a). Under light-stress conditions, a certain portion of ^1^O_2_ evades the ^1^O_2_ quenchers in PSII and diffuses into the thylakoid membrane, where it leads to lipid peroxidation. As a preventive measure, both tocopherol (Kruk et al., 2005) and PQ (Kruk and Trebst, 2008; Yadav et al., 2010), present in the thylakoid membranes, can detoxify ^1^O_2_. In the stroma, ascorbate can also scavenge ^1^O_2_ if it is released from the thylakoid membranes (Bisby et al., 1999).

**Figure 2:**
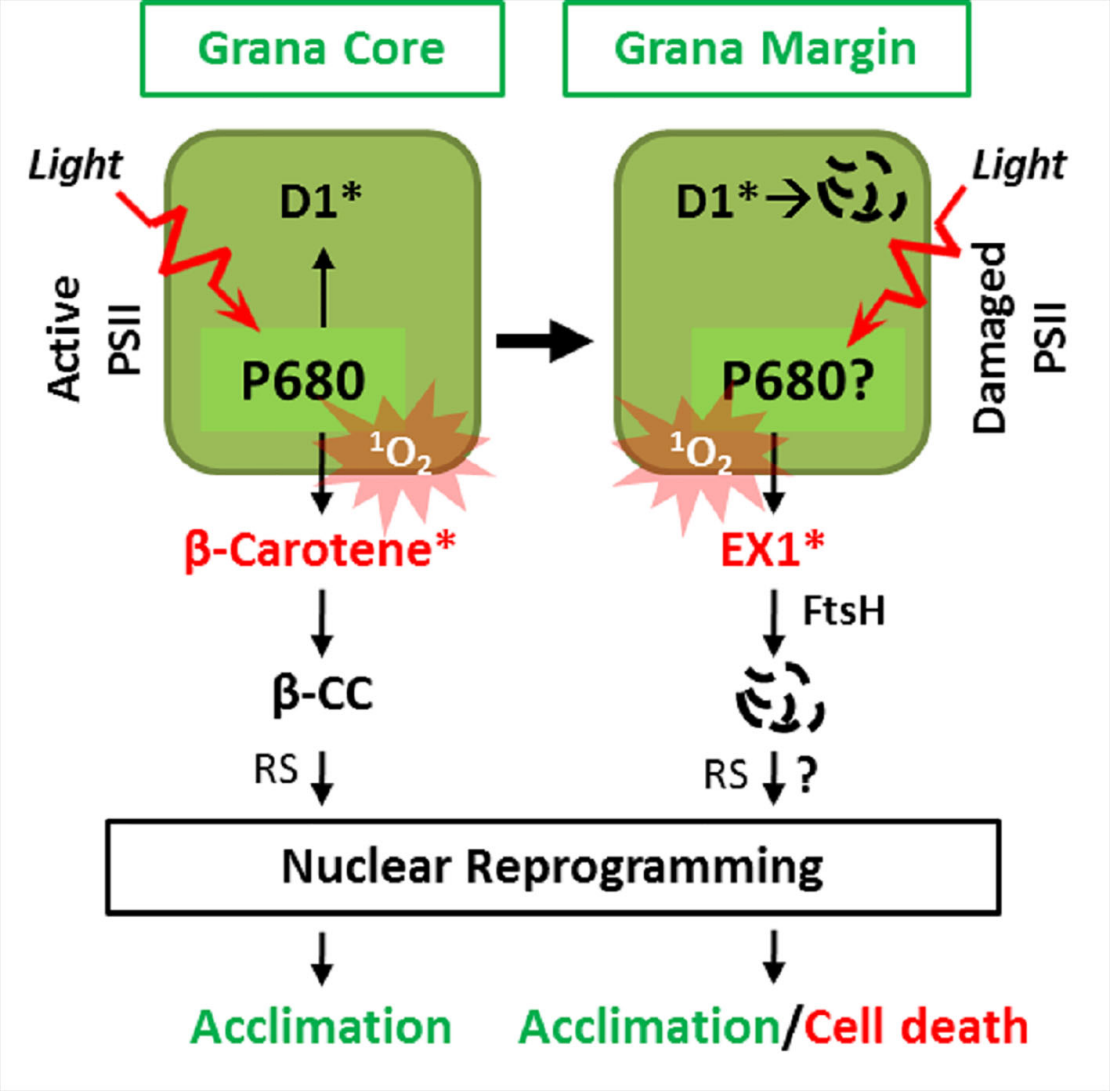
Two spatially separated ^1^O_2_ sensors and cognate retrograde signaling pathways. ^1^O_2_ generated in the GC by the excited triplet state of P680 damages PSII RC, facilitating its repair in the GM. Under light stress conditions, the enhanced levels of ^1^O_2_ result in the accumulation of β-cyclocitral (β-CC), a volatile oxidative product of β-carotene, which mediates retrograde signaling to activate genes involved in detoxification, photoprotection, and acclimation. In the GM, the unbound free chlorophyll molecules released during disassembly/reassembly of PSII or by *de novo* synthesis may generate ^1^O_2_. The EX1 protein residing in the GM then senses the ^1^O_2_ via its SOS domain and initiates ^1^O_2_ signaling through the coordination of the FtsH protease. The EX1 proteolysis by FtsH appears to be essential for inducing changes in nuclear gene expression priming acclimation or cell death. The genuine signaling molecule(s) and the downstream signaling components involved in EX1-mediated signaling remain to be elucidated. *Denotes oxidized state.

Despite the efficient quenching by β-carotene, some amount of ^1^O_2_ diffuses within the PSII RC leading to the irreversible modification of PSII RC proteins, especially D1 (Telfer, 2002; Vass and Cser, 2009). The irreversible modification hinders the electron transfer function of D1 and subsequently impairs the PSII activity (Aro et al., 1993) (**Figure 2**). The damaged PSII undergoes a repair process in the non-appressed grana called grana margin (GM) (Aro et al., 1993). The PSII repair involves the monomerization of the PSII dimer, followed by the migration of the PSII monomer from the GC to the GM, disassembly, degradation, and *de novo* synthesis of D1, insertion of the newly synthesized D1 protein into the thylakoid membrane concomitantly with the assembly of other components of PSII, migration of the PSII monomer to the grana core, and finally dimerization (Aro et al., 1993; Hakala et al., 2005). The GM-localized membrane-bound hexameric FtsH metalloprotease plays a crucial role in PSII proteostasis by degrading damaged D1 proteins (Kato et al., 2009; Malnoë et al., 2014). Since D1 binds chlorophyll in the PSII, chlorophyll molecules probably become unbound during disassembly allowing the efficient degradation of D1. Consistently, during the *de novo* synthesis of the D1 protein, the nascent polypeptide chain is directly inserted into the D1-depleted PSII along with recycled or newly synthesized chlorophyll molecules. Collectively, the ^1^O_2_-driven oxidative modification of D1 seems to underline its turnover (Aro et al., 1993; Hakala et al., 2005; Kato et al., 2009; Malnoë et al., 2014). Besides D1, the D2 protein of the PSII RC also exhibits a decline upon a ^1^O_2_ burst, indicating probable photodamage of D2 by ^1^O_2_ (Koivuniemi et al., 1995; Jansen et al., 1999; Edelman and Mattoo, 2008). In accordance, recent studies have demonstrated that the oxidative modification is not limited to D1, but rather all PSII RC proteins including D2, CP43, and CP47 get oxidized by ^1^O_2_ (Dreaden Kasson et al., 2012; Dogra et al., 2019a; Dogra et al., 2019b; Duan et al., 2019). Though these oxidative modifications might be linked with the turnover, the precise relevance of these modifications in PSII proteins needs to be investigated.

## β-carotene Also Act as a ^1^O_2_ Sensor in the GC to Initiate RS

The primary function of ß-carotene residing in the PSII RC is to scavenge ^1^O_2_ to prevent PSII damage (Foote and Denny, 1968; Ramel et al., 2012b). This scavenging, however, leads to its non-enzymatic oxidative modification into aldehydes and endo-peroxides (Foote and Denny, 1968; Ramel et al., 2012b). Under normal light conditions, the ^1^O_2_-specific endo-peroxides of ß-carotene such as ß-cyclocitral (ß-CC), Dihydroactinidiolide (dhA), and β-ionone are only detectable at negligible levels. However, under high light (HL), these oxidation products accumulate to significant levels (Ramel et al., 2012b). These endo-peroxides accumulate in proportion to the extent of PSII photoinhibition, which supports the notion that the PSII RC is the primary site of ^1^O_2_ generation under excess light conditions (Ramel et al., 2012a). Interestingly, these endo-peroxides of β-carotene are highly reactive, volatile, and electrophilic compounds, thus referred to as reactive electrophile species (RES) (Ramel et al., 2012b). Recent studies in Arabidopsis wild-type and *chlorina 1* (*ch1*) mutant plants revealed that ß-CC and to some extent dHA act as ^1^O_2_-signaling molecules (Ramel et al., 2012b; Ramel et al., 2013; Shumbe et al., 2014). Exogenous application of these RES compounds activates a plant acclimation response which mitigates the HL-induced photo-oxidative damages including lipid peroxidation. Pretreatment with ß-CC induces the expression of genes involved in photo-protection to prevent lipid peroxidation, leaf bleaching, and to maintain the photochemical activity upon subsequent exposure to HL (Ramel et al., 2012b). ß-CC induces a specific subset of genes, referred to as ^1^O_2_-responsive genes (SORGs), which are quite distinct from those induced by H_2_O_2_ (Ramel et al., 2012b) (**Figure 2**). As compared to ß-CC, dhA appears to induce a small subset of these SORGs (Shumbe et al., 2014), indicating that it is not a significant contributor in ß-carotene-mediated RS.

The β-CC, being volatile, probably reaches the cytosol and/or the nucleus where it induces SORGs *via* some signaling components. An earlier genetic screen in the unicellular green alga *Chlamydomonas reinhardtii* has unveiled the small zinc finger protein Methylene Blue Sensitivity 1 (MBS1), localized both in the cytosol and the nucleus, as a probable downstream component of ß-CC-mediated ^1^O_2_ signaling (Shao et al., 2013). Consistently, Arabidopsis *mbs1* mutant plants are not able to acclimate after pretreatment with β-CC to the lethal dose of HL stress (Shumbe et al., 2017). It was also shown that the MBS1 protein was required for the expression of a subset of β-CC-induced SORGs implicated in photo-acclimation (Shumbe et al., 2017). However, the precise mechanism of how MBS1 drives the gene expression remains unclear. A recent study reported that β-CC activates the TGAII/scarecrow like-14 (SCL14) transcription factors to induce detoxification-related genes required for the acclimation towards HL stress in Arabidopsis (D’Alessandro et al., 2018). Interestingly, the β-CC-mediated activation of TGAII/SCL14 TFs and downstream gene expression is independent of MBS1 (D’Alessandro et al., 2018), which suggests the existence of multiple components involved in this retrograde signaling operating in a spatio-temporal manner. Collectively, these observations conclude that the ^1^O_2_ quencher β-carotene also act as a ^1^O_2_ sensor in the PSII RC in the GC (**Figure 2**).

## The Grana Margin-Associated ^1^O_2_ Sensor Executer1

Klaus Apel and his co-workers first demonstrated the signaling role of ^1^O_2_ by utilizing the Arabidopsis *fluorescent* (*flu*) mutant (Meskauskiene et al., 2001). The *flu* mutant plants conditionally generate ^1^O_2_ in chloroplasts upon a dark-to-light shift (Meskauskiene et al., 2001). In the dark, the FLU protein negatively regulates the accumulation of protochlorophyllide (Pchlide), a precursor of chlorophyll. Therefore, *flu* mutant plants initially grown under continuous light (permissive) conditions overaccumulate free Pchlide (non-protein-bound form) in the dark, which upon re-illumination acts as a potent photosensitizer (like ^3^Chl*), leading to the ^1^O_2_ generation (Meskauskiene et al., 2001). This intriguing characteristic of the *flu* mutant enabled the discovery that ^1^O_2_ rapidly induces nuclear gene expression changes, which precede cell death in seedlings and growth inhibition in mature plants (Meskauskiene et al., 2001; op den Camp et al., 2003). Hence, the *flu* mutant is now used as a bio-tool for investigating the ^1^O_2_-mediated signaling and the mechanisms of chloroplast-mediated stress responses including cell death and growth inhibition (Meskauskiene et al., 2001; op den Camp et al., 2003; Wagner et al., 2004; Lee et al., 2007). Using this biotool, the genetic basis of ^1^O_2_-mediated signaling has been illustrated: the nuclear-encoded chloroplast protein EXECUTER 1 (EX1) mediates ^1^O_2_-triggered nuclear gene expression changes, cell death, and growth inhibition (Wagner et al., 2004; Kim et al., 2012). Loss of EX1 significantly abolishes the ^1^O_2_-induced stress responses in the *flu* mutant plants (Wagner et al., 2004; Lee et al., 2007). Also, EXECUTER2 (EX2), a close homolog of EX1, participates in the EX1-mediated ^1^O_2_ signaling as a putative modulator (Lee et al., 2007). Loss of function of both EX1 and EX2 almost completely abrogates the ^1^O_2_-triggered stress responses in the *flu* mutant upon a dark-to-light shift (Lee et al., 2007) as well as in wild-type plants exposed to moderate light stress (Kim et al., 2012). Besides activating cell death, EX1 and EX2 are also involved in local and systemic gene expression changes leading to acclimation toward HL stress (Carmody et al., 2016). Interestingly, the EX1-mediated signaling was found to be distinct and independent of the one mediated by ß-carotene (Ramel et al., 2013). This finding raises an important question regarding the reason for the existence of two independent ^1^O_2_-signaling pathways.

Recent studies aiming to understand the mechanism of EX1-mediated ^1^O_2_ signaling have provided the first hint for understanding these two signaling systems. It appears that EX1 mostly localizes in the GMs. This result indicates that EX1 resides away from the active PSII RC in the GC, the primary site of ^1^O_2_ generation. In the GMs, EX1 associates with PSII RC proteins (undergoing repair) and the PSII repair machinery (**Figure 2**; Wang et al., 2016). The nearly exclusive localization of EX1 in the GM not only resolves the vagueness of the presence of two independent ^1^O_2_-triggered retrograde signaling pathways but also suggests the presence of an additional site of ^1^O_2_ generation (Wang et al., 2016; Dogra et al., 2018; Foyer, 2018) (**Figure 2**). The idea regarding the alternative site of ^1^O_2_ generation (i.e., GM) is in line with an earlier study showing that ^1^O_2_ may also compromise the *de novo* synthesis of D1, which takes place at the GM, by directly targeting the PSII repair machinery as observed for the chloroplast elongation factor G (CpEF-G) in *Synechocystis* sp. strain PCC6803 (Nishiyama et al., 2004). It is noteworthy that the oxidation of CpEF-G by ^1^O_2_ in higher plants has not been reported to date.

Nevertheless, given the very short half-life (~200 ns) (Gorman and Rodgers, 1992) and imminent reactivity (Krieger-Liszkay, 2005), it is almost impossible that ^1^O_2_ generated at PSII in the GC travels to the GM to oxidize the PSII repair machinery. Therefore, it is likely that the non-appressed thylakoid region may also generate ^1^O_2_, perhaps during the PSII repair (Dogra et al., 2018). Another explanation might be that other reactive species with a more extended lifespan move from the GC to the GM to oxidize the PSII repair machinery. Although it remains to be elucidated, one may suppose that perturbations in the PSII repair may lead to the release of ^1^O_2_ by tetrapyrrole molecules required for PSII reassembly. In fact, it was shown that EX1 also interacts with enzymes involved in chlorophyll biosynthesis (Wang et al., 2016).

## Oxidative Modification of Executer1 is Essential to Mediate ^1^O_2_ Signaling

Aiming to understand how EX1 mediates ^1^O_2_ signaling, Wang et al. (2016) realized that EX1 undergoes degradation upon ^1^O_2_ burst. This observation indicated that EX1 might undergo a post-translational modification *via* ^1^O_2,_ and its associated protease may coordinate ^1^O_2_ signaling. Accordingly, EX1 was found to associate with the FtsH protease in the GM (Wang et al., 2016). Given that the FtsH protease complex is involved in PSII repair (Kato and Sakamoto, 2009) and that EX1 is associated with PSII proteins in the GM, EX1 might also affect PSII repair. However, neither the presence nor absence of EX1 did modulate the PSII repair (Wang et al., 2016). Nonetheless, a resulting reverse genetic approach revealed that EX1 proteins undergo FtsH-dependent proteolysis, which turned out to be an essential step in initiating ^1^O_2_ signaling (**Figure 2**). The loss of FtsH2, the major subunit of the FtsH protease, substantially compromises ^1^O_2_ signaling in the *flu* mutant (Wang et al., 2016; Dogra et al., 2017; Dogra et al., 2019b) (**Figure 2**). Based on these observations, it was postulated that FtsH might recognize EX1 as a substrate probably upon its oxidation or conformational changes in response to ^1^O_2_. Consistently, it was revealed that EX1 undergoes an oxidative post-translational modification (Oxi-PTM) at a specific tryptophan residue (Trp643) located in the domain of unknown function 3506 (DUF3506) upon exposure to ^1^O_2_. This modification is indispensable for its degradation and the subsequent activation of RS. As Trp643 is present in the DUF3506 and this domain is vital for degradation, it is now dubbed as singlet oxygen senor (SOS) domain, and EX1 protein is designated as a ^1^O_2_ sensor.

The EX1 proteolysis *via* the FtsH protease may release a yet unknown signaling molecule. A small EX1 proteolytic peptide per se may activate the ^1^O_2_-triggered signaling cascade (**Figure 2**). Considering that EX2 also possess DUF3506, it is very likely that it might also undergo oxidative modification and the subsequent degradation. Further research addressing the possible release of a small peptide or signaling molecule correlated with EX1 degradation and the role of EX2 in EX1-mediated signaling would provide mechanistic insights into this retrograde signaling.

## Thylakoid Lipids Can Also Activate ^1^O_2_ Signaling *via* Their Oxidative Products

Besides proteins and carotenoids in PSII, ^1^O_2_ also targets thylakoid lipids. Oxidation of the PUFAs in the lipids results in the accumulation of oxidized lipid products, also called oxylipins, in the thylakoid membrane. Many of these oxylipins are electrophilic due to their α,β-unsaturated carbonyl groups allowing them to react with electron-rich atoms in biological molecules. These lipid-derived RES include 12-oxo-phytodienoic acid (OPDA), phytoprostanes, aldehydes, and ketones (Imbusch and Mueller, 2000; Gobel et al., 2002; Mano, 2012). Some of these RES can alter the expression of nuclear genes to activate stress responses, including detoxification, defense, and cell death (Weber et al., 2004; Sattler et al., 2006; Mueller et al., 2008; Farmer and Mueller, 2013). The RES-driven nuclear gene expression changes overlap significantly with those induced by β-CC-mediated RS in Arabidopsis.

In green alga *Chlamydomonas reinhardtii*, pretreatment with a sub-lethal dose of ^1^O_2_ induced rapid nuclear gene expression changes, conferring acclimation towards a subsequent challenge with a lethal dose of ^1^O_2_ (Ledford et al., 2007). This pre-acclimation resulted in the significant upregulation of detoxification-related genes, such as *Glutathione Peroxidase* (*GPX5/GPXH*) and a *Glutathione-S-Transferase* (*GSTS1*), to sustain photo-oxidative stress (Ledford et al., 2007). The set of genes induced were similar to those induced by β-CC mediated signaling. However, the levels of β-CC were almost unchanged, whereas the lipid-derived α,β-unsaturated aldehydes, including 2-propenal (also called acrolein), hexenal, and malonaldehyde (MDA) were elevated in response to HL in Chlamydomonas (Roach et al., 2017), suggesting possible participation of these lipid-derived RES in this signaling. Notably, these lipid-derived RES tend to accumulate in response to ^1^O_2_ stress not only in Chlamydomonas but also in plant leaves (Mano et al., 2010). Among these RES, acrolein comparatively shows higher accumulation. Although acrolein imposes toxicity to the plants by directly damaging photosynthetic apparatus (Mano et al., 2009), its exogenous treatment at comparatively lower levels can induce the expression of RES-responsive detoxification genes, including *GST1* (Roach et al., 2017; Roach et al., 2018). Recent findings show that carotene-derived RES β-CC activates SCL14/TGAII and NAC TFs to activate downstream detoxification system in Arabidopsis (D’Alessandro et al., 2018), on the other hand, lipid-derived RES activate *Singlet Oxygen Resistant 1* (*SOR1*), a bZIP10 transcription factor, to drive the expression of acclimation related genes in Chlamydomonas (Fischer et al., 2012). According to these recent findings, the role of the lipid-derived RES is apparent in ^1^O_2_ signaling, though the precise mechanism of their perception and activation of downstream signaling remains to be elucidated. Because many RES molecules, both carotenoid- and lipid-derived, are generated simultaneously upon exposure to light stress, linking a RES to a particular RS pathway remains mostly unexplored.

## Concluding Remark

The ^1^O_2_ is generated as a byproduct of photosynthesis. In addition to its well-known damaging effect, at sub-lethal levels, ^1^O_2_ seems to trigger distinct retrograde signaling pathways *via* oxidative modification of carotenoids, proteins, and lipids to activate multiple stress responses (Ledford et al., 2007; Kim et al., 2008; Triantaphylides and Havaux, 2009; Kim and Apel, 2013; Zhang et al., 2014; Zhu, 2016; Dogra et al., 2018). Recent studies have established that the thylakoid membranes are equipped with distinct ^1^O_2_ sensors: β-carotene located in the GC and EX1 proteins localized in the GM, both of which undergo oxidative modifications to mediate independent ^1^O_2_-signaling pathways (Ramel et al., 2012b; Dogra et al., 2019b). These studies also suggested that, besides the active PSII in the GC, the GM may also generate ^1^O_2_, perhaps during PSII repair (Wang et al., 2016; Dogra et al., 2018; Foyer, 2018). While the source of ^1^O_2_ generation in the GC is well understood, it is unclear how the GM generates ^1^O_2_. In these regards, further investigation on ^1^O_2_ sensors would provide insights into the source of EX1 oxidation in the GM and the reason for the presence of two distinct ^1^O_2_ signaling pathways. Although the role of thylakoids lipids is emerging as putative sensors of ^1^O_2_, the precise mechanism by which the lipid peroxidation-mediated signaling activates the retrograde signaling need to be investigated.

## Author Contributions

VD and CK wrote the manuscript.

## Funding

This work was supported by the Strategic Priority Research Program from the Chinese Academy of Sciences (Grant No. XDB27040102). VD acknowledge the President’s International Fellowship Initiative (PIFI) postdoctoral fellowship from the Chinese Academy of Sciences (No. 2019PB0066).

## Conflict of Interest

The authors declare that the research was conducted in the absence of any commercial or financial relationships that could be construed as a potential conflict of interest.
